# Spontaneous Biloma With a Hepatic Mass

**DOI:** 10.7759/cureus.40249

**Published:** 2023-06-11

**Authors:** Venu M Ganipisetti, Pratyusha Bollimunta, Natasha Dudiki

**Affiliations:** 1 Hospital Medicine, Presbyterian Hospital, Albuquerque, USA; 2 Pulmonary and Critical Care, Indiana University Health Ball Memorial Hospital, Muncie, USA

**Keywords:** biloma, gi cancer, cancer, gi malignancy, hepatic mass, spontaneous biloma

## Abstract

We report a case of an 86-year-old Hispanic male who presented with generalized itching and jaundice. Computed tomography (CT) imaging revealed a hepatic mass and an extensive spontaneous biloma, a condition rarely associated with malignancy. Subsequent biopsy of the mass confirmed moderately differentiated adenocarcinoma of the pancreaticobiliary tract. The patient underwent successful percutaneous drainage of the biloma and was discharged with a plan for further outpatient management. This case study highlights a rare manifestation of spontaneous biloma related to malignancy, broadening the clinical understanding of its association with malignancy, diagnosis, and management.

## Introduction

Biloma, an encapsulated extra biliary collection of bile, is a rare condition typically associated with traumatic or iatrogenic injury to the biliary ducts. Less common are spontaneous bilomas, often linked to choledocholithiasis and very rarely related to malignancies. This report presents an intriguing case of an 86-year-old male diagnosed with a hepatic mass and an extensive biloma, representing a rare manifestation of malignancy-associated spontaneous biloma.

## Case presentation

An 86-year-old Hispanic male with a history of hypertension, hyperlipidemia, and stage IIIb chronic kidney disease (baseline creatinine: 1.7 mg/dL), presented to urgent care with a two-week history of generalized itching. The patient denied weight loss, abdominal pain, nausea, or vomiting. He had a remote smoking history which he quit about 50 years ago, and he quit alcohol 30 years ago. Home medications included atenolol, lisinopril, and atorvastatin. He had no known allergies. He was afebrile and hemodynamically stable. He had jaundice with scleral icterus. His abdomen was soft, non-tender, and non-distended. Otherwise, the physical exam was unremarkable.

**Figure 1:**
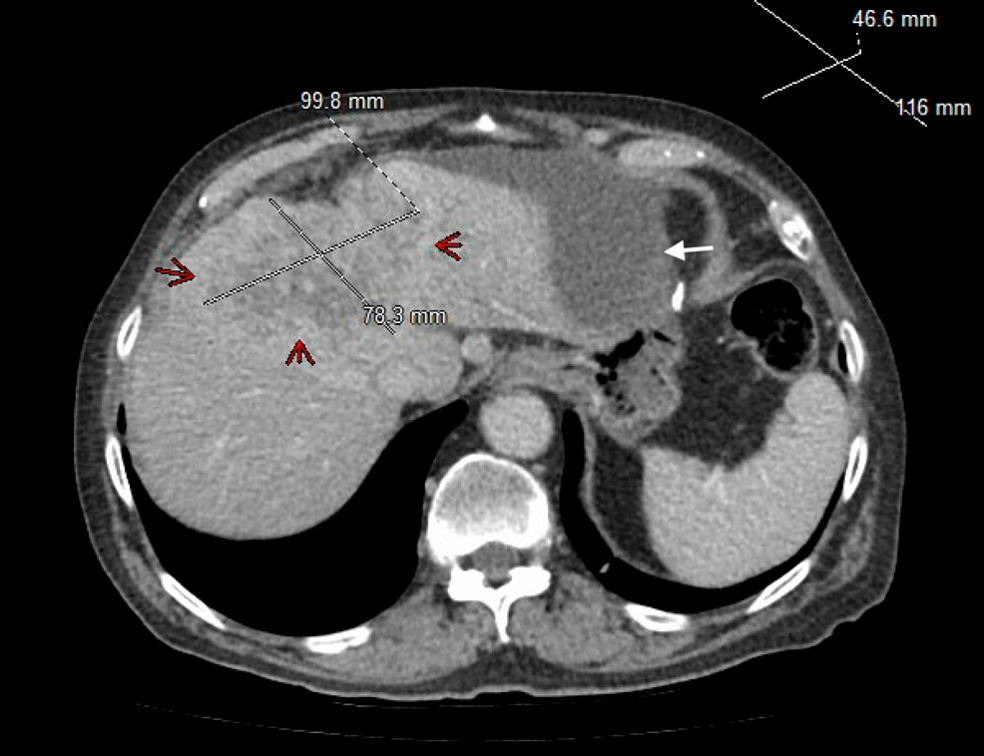
Red arrows pointing to the hepatic mass and the white arrow pointing to the biloma.

**Figure 2:**
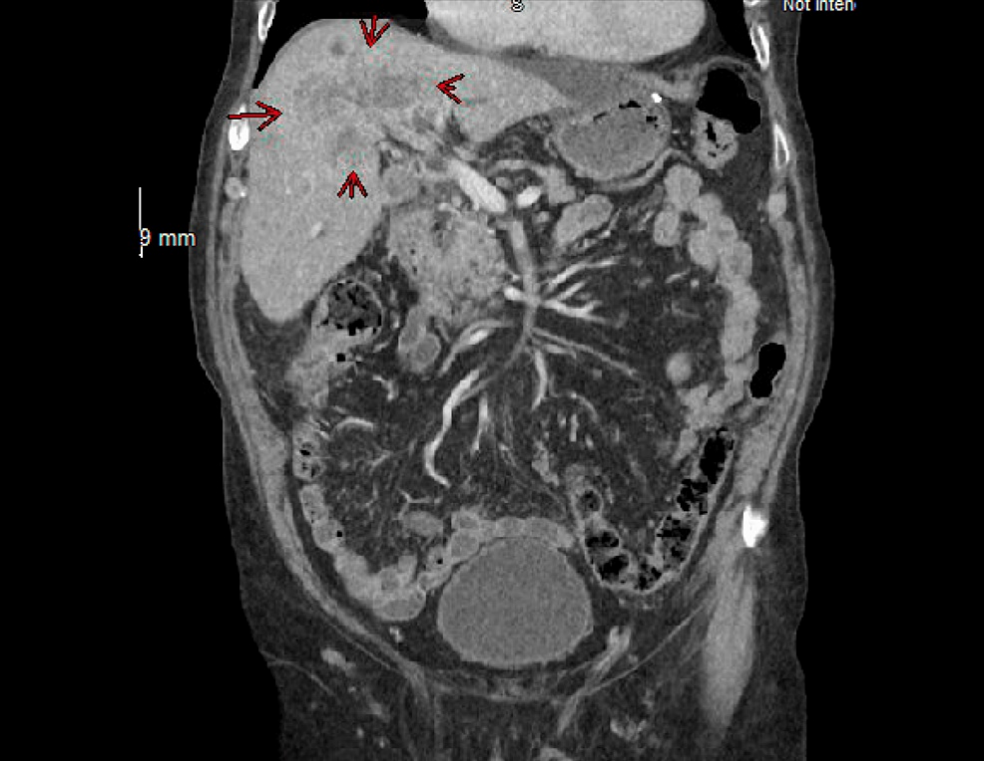
Red arrows pointing at the hepatic mass.

**Figure 3:**
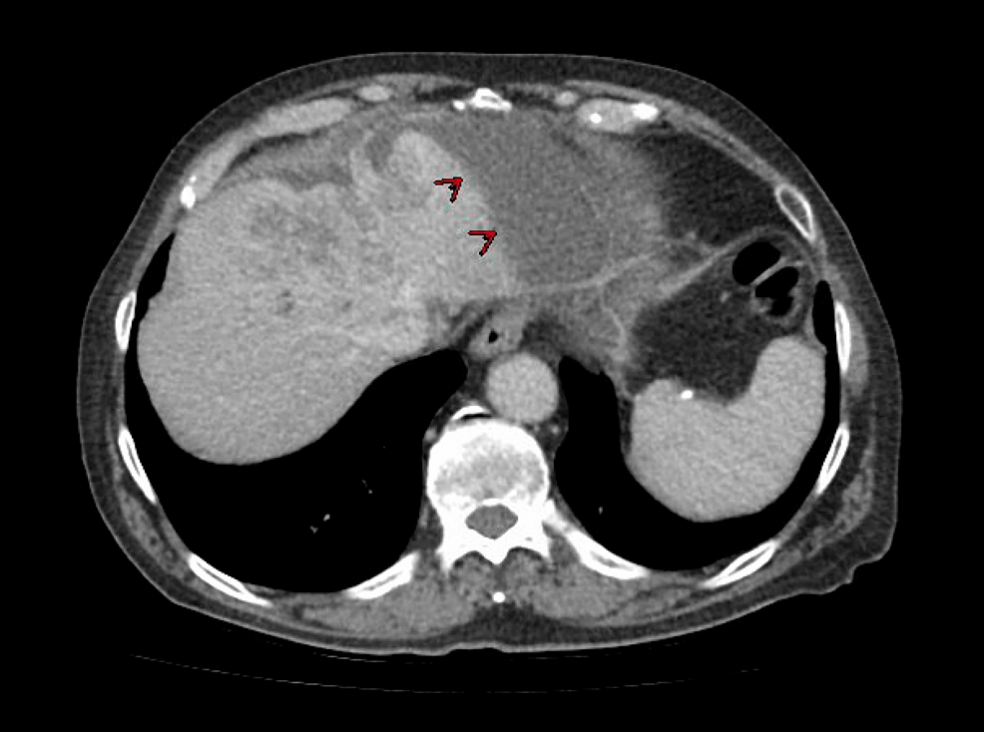
Red arrows are pointing at the biloma.

**Figure 4:**
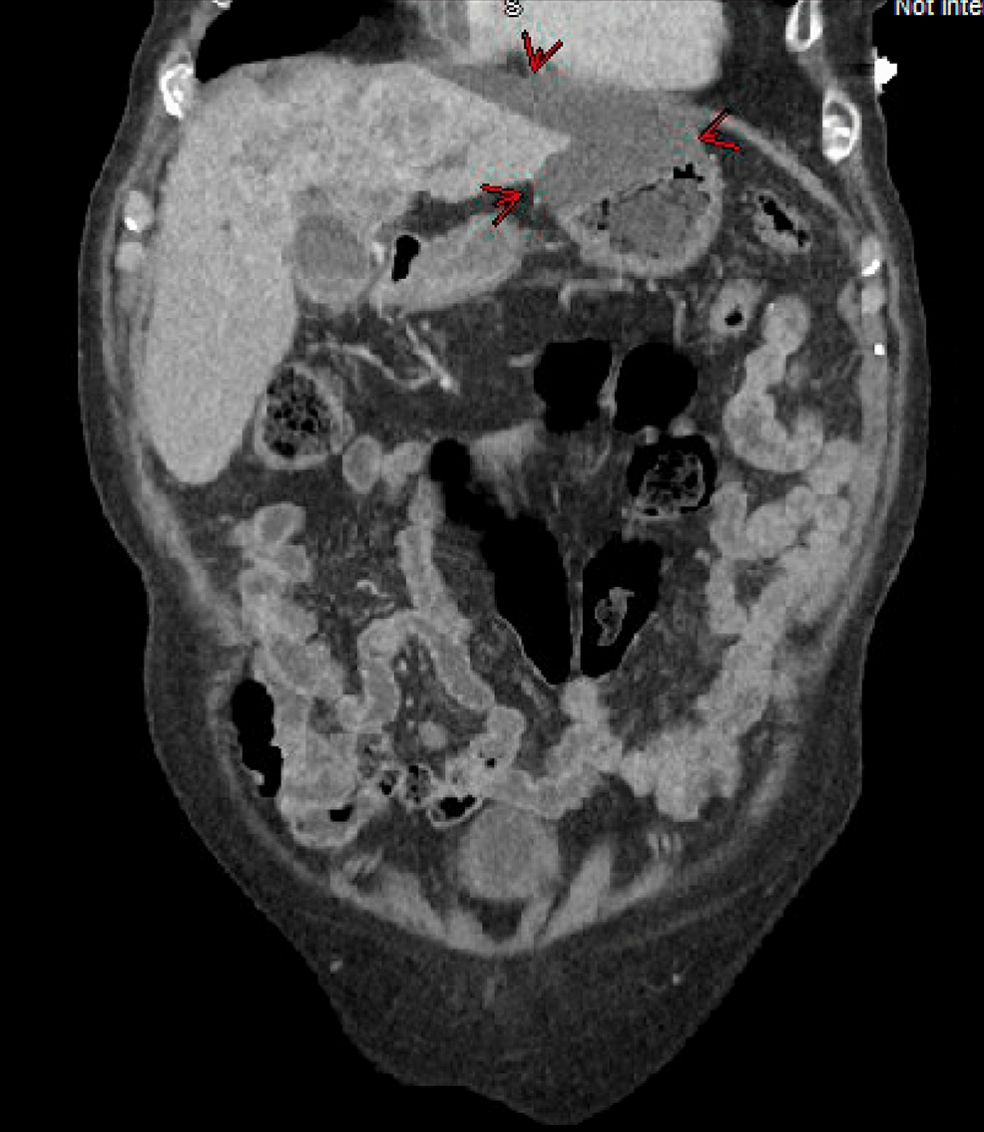
Red arrows are pointing at the biloma.

The leukocyte count was normal. Creatinine was 1.8 mg/dL (normal: 0.7-1.35 mg/dL). Liver enzymes were abnormal. Total bilirubin was 11 mg/dL (0.0-1.2 mg/dL), with direct bilirubin of 8.7 mg/dL, indirect bilirubin of 2.3 mg/dL, alkaline phosphatase at 596 U/L (normal: 40-129 U/L), aspartate transaminase (AST) at 95 U/L (10-50 U/L), and alanine transaminase (ALT) at 25 U/L (10-55 U/L). Lipase was normal, and urinalysis was negative. Viral hepatitis panel, flu, respiratory syncytial virus (RSV), and COVID-19 tests were negative. Computed tomography (CT) abdomen and pelvis showed an infiltrating hepatic mass measuring 10 x 7.8 x 7.7 cm with mild intrahepatic biliary ductal dilatation suspicious for intrahepatic cholangiocarcinoma (Figures 1, 2). He was noted to have an extensive biloma developing along the left lobe of the liver, extending towards the stomach, measuring nearly 12 cm in length and up to 4.7 cm in diameter (Figures 3, 4). Several small, mildly enlarged lymph nodes in porta hepatis were noted, suspicious for metastatic nodes. There was no evidence of cholelithiasis on imaging. The patient was admitted for further management.

We started the patient on ceftriaxone and metronidazole for empiric coverage for the possibility of an infected biloma. He underwent a biopsy of the liver mass and drainage of the biloma (with a drain left in place) by Interventional Radiology. The fluid sample from the biloma had the appearance of bile and culture resulted in no growth. Antibiotics were therefore discontinued. Cancer antigen 19-9 (CA 19-9) level, cancer antigen 125 (CA 125), carcinoembryonic antigen (CEA), and alpha-fetoprotein (AFP) were not elevated. A biopsy of the liver mass showed moderately differentiated adenocarcinoma compatible with pancreaticobiliary tract primary. CT imaging of the chest did not show any obvious evidence of metastasis. Given the enlarged lymph nodes in porta hepatis suggesting lymph node metastasis, age, and chronic kidney disease, surgical resection was not felt to be an ideal option. The patient was referred to radiation oncology, with plans for outpatient follow-up with oncology to discuss further management options. By day 12, drainage from the biloma stopped. On day 15, a diagnostic abscessogram demonstrated no significant residual fluid in the cavity and no fistulous communication to the adjacent bowel. The drain was removed. The patient was discharged in stable condition with plans to follow up with radiation oncology, palliative care, and medical oncology as an outpatient.

## Discussion

Biloma is an encapsulated extra biliary collection of bile. It is most commonly caused by traumatic or iatrogenic injury to biliary ducts. Rarely, bilomas can develop in the absence of trauma or surgical manipulation, and these are referred to as spontaneous bilomas. The most common cause of spontaneous biloma is choledocholithiasis [1,2]. In previous reports, it was also seen relation to cholecystolithiasis, hepaticolithiasis, acute cholecystitis, hepatic infarction, hepatic abscess, nephrotic syndrome, sickle cell disease, tuberculosis, obstructive jaundice and often idiopathic etiologies [​​1,2]. Malignancy causing spontaneous bilomas is exceedingly rare; to our knowledge, only 10 cases have been reported so far. Bilomas may be located intra or extrahepatic.

Since the introduction of the term biloma in 1979, very few cases of spontaneous bilomas have been reported. In 1998 Fujiwara et al. noted 25 cases (12 male, 13 female) of spontaneous biloma in literature during 1979-1997; two out of the 25 cases were secondary to malignancy (cancer of the biliary tree) [2]. In 2020, Suzuki et al. identified additional 28 cases (15 male, 13 female) of spontaneous biloma in a literature search from 2001 to 2019, and only four out of 28 cases were secondary to malignancy [1]. We performed a literature review in Pubmed using the search strategy "spontaneous biloma AND malignancy." We identified eight reported cases of malignancy-associated spontaneous bilomas by eight authors [1,3-9] in addition to the two identified cases by Fujiwara et al. [2].

Mechanisms in the formation of spontaneous biloma include biliary ductal necrosis secondary to impacted gallstone, rupture of diverticulum or cyst or obstruction by a mass gallstone, or spasm of the sphincter of Oddi causing increased intraductal pressure and subsequent weakening of the bile duct wall [2,10].

Symptoms can be nonspecific [1]. The most common symptoms include right upper quadrant pain, nausea, vomiting, fevers, chills, and icterus [1]. Our patient did not have any pain, nausea, or vomiting. The only symptoms in our case were itching and jaundice.

Bilomas can progress to cause biliary peritonitis, can get infected, and lead to abscess formation, sepsis, septic shock, respiratory failure, and cholestasis secondary to impingement on the biliary tree [11]. Hence, these need prompt treatment.

On lab testing, bilomas do not present with typical abnormalities. Patients may show elevated inflammatory markers, especially in the case of infected bilomas, and show abnormal liver enzymes such as in our case. Blood cultures may be positive in infected bilomas. Diagnosis is based on radiological findings noted on CT imaging. Ultrasound (US) findings of a hypo-anechoic fluid collection with well-defined margins are suggestive of biloma, but are non-specific and need confirmation with contrast-enhanced CT [12]. Magnetic resonance imaging (MRI) and hepatobiliary iminodiacetic acid (HIDA) scans can add further information but are not required to make a diagnosis.

The primary treatment for biloma is percutaneous drainage of the fluid collection. However, depending on the etiology of the biloma, further surgical interventions would be required, such as endoscopic retrograde cholangiopancreaticography (ERCP) and cholecystectomy for choledocholithiasis, surgical resection for masses, etc. In cases of persistent biliary leak, ERCP and stenting can be utilized [13]. Treatment with antibiotics is required when blood and biliary fluid cultures are positive. Our patient was treated with percutaneous drain placement by interventional radiology, and in 12 days, drainage spontaneously stopped, and the drain was removed. Most bilomas respond well to percutaneous drainage and do not require repair of the disrupted biliary tree. Recurrence is rare.

## Conclusions

This case illuminates a rare instance of malignancy-associated spontaneous biloma, with a hepatic mass suggestive of adenocarcinoma of the pancreaticobiliary tract as the underlying etiology. We report this case to broaden our clinical understanding of the possible etiologies of spontaneous bilomas, underlining the importance of considering malignancy in the differential diagnosis and emphasizing the critical role of biopsy and radiological investigations in such cases. The response to treatment further reinforces the effectiveness of percutaneous drainage as a treatment modality for biloma. However, each patient should be managed based on individual circumstances, including the underlying cause of the biloma and the patient's overall health status.
